# Correcting for Optimistic Prediction in Small Data Sets

**DOI:** 10.1093/aje/kwu140

**Published:** 2014-06-24

**Authors:** Gordon C. S. Smith, Shaun R. Seaman, Angela M. Wood, Patrick Royston, Ian R. White

**Keywords:** logistic models, models, statistical, multivariate analysis, receiver operating characteristic curve

## Abstract

The *C* statistic is a commonly reported measure of screening test performance. Optimistic estimation of the *C* statistic is a frequent problem because of overfitting of statistical models in small data sets, and methods exist to correct for this issue. However, many studies do not use such methods, and those that do correct for optimism use diverse methods, some of which are known to be biased. We used clinical data sets (United Kingdom Down syndrome screening data from Glasgow (1991–2003), Edinburgh (1999–2003), and Cambridge (1990–2006), as well as Scottish national pregnancy discharge data (2004–2007)) to evaluate different approaches to adjustment for optimism. We found that sample splitting, cross-validation without replication, and leave-1-out cross-validation produced optimism-adjusted estimates of the *C* statistic that were biased and/or associated with greater absolute error than other available methods. Cross-validation with replication, bootstrapping, and a new method (leave-pair-out cross-validation) all generated unbiased optimism-adjusted estimates of the *C* statistic and had similar absolute errors in the clinical data set. Larger simulation studies confirmed that all 3 methods performed similarly with 10 or more events per variable, or when the *C* statistic was 0.9 or greater. However, with lower events per variable or lower *C* statistics, bootstrapping tended to be optimistic but with lower absolute and mean squared errors than both methods of cross-validation.

The ability to predict outcomes (e.g., disease, death, relapse) is important in many areas of medicine, such as population screening and assessment of prognosis and response to treatment. With expansion in the technology around biomarker development (including genomics, proteomics, and metabolomics), there is increasing capacity to generate multiple biomarkers for a given condition (1). Moreover, increasingly sophisticated biomedical technology allows more accurate phenotyping of adverse outcomes, leading to analyses of smaller subgroups of disease (2). Consequently, many studies end up evaluating multiple potential predictors using data sets with relatively small numbers of cases. Analysis of multiple markers generally involves fitting a statistical model to the data. Because models are generated to provide the best fit for the available data, there is the potential that a model will be overfitted and, hence, provide an optimistic assessment of the predictive ability, quantified here by the *C* statistic (also known as the area under the receiver operating characteristic curve).

It is generally recognized that external validation of a model is required. Some studies split samples into 2, but then the validation is not truly external. Moreover, when external data are studied for validation, the analysis may be weakened by small sample size. Hence, it is important in the initial internal evaluation of a candidate model to correct estimates of predictive ability for optimism. A number of statistical methods have been proposed to address this (3). Commonly used approaches include sample splitting, cross-validation, and bootstrapping. Variants of cross-validation include its use with or without replication, leave-1-out cross-validation, and a more recently described development of the approach, called leave-pair-out cross-validation. The aim of the present study was to compare these approaches to correcting for optimism.

## METHODS

### Overview

We evaluated different methods of correcting the *C* statistic using similar approaches to those of Steyerberg et al. (4). We obtained 2 large clinical data sets. In each of these, we divided the large data set into multiple distinct small data sets (without resampling), each containing approximately 5 events per variable (EPV) (which was, intentionally, well below the generally recommended EPV of 10 (3)) and approximately 4 controls per case. We reduced the number of controls to better simulate the type of study in which optimism is an issue. We fitted models to each of the small data sets and assessed their predictive ability in the rest of the large data set (i.e., all of the other small data sets pooled together), which we used as a “gold standard.” We then compared the performance of each method by comparing the optimism-adjusted *C* statistic estimated from the small data set with the gold standard. The use of a case-control design was simply for convenience, and the methods we used are appropriate for any application of logistic regression.

### Clinical data

The first source of data was United Kingdom Down syndrome screening laboratories located in Glasgow (1991–2003), Edinburgh (1999–2003), and Cambridge (1990–2006) (5). All records that included data on maternal age, maternal serum level of α-fetoprotein, and maternal serum human chorionic gonadotropin were used. The pooled data from the 3 centers included 466,309 records and 785 cases of Down syndrome (0.17%). The 3 predictors evaluated were maternal age, maternal serum α-fetoprotein, and maternal serum human chorionic gonadotropin (both expressed as the log_10_ of the multiple of the median for gestational age, as is conventional (6)). We randomly created 50 small data sets each containing 15 or 16 cases and 66 or 67 controls. The second source of data was the Scottish Morbidity Record 2, a national registry of pregnancy discharge data, from which we selected records from 2004 to 2007 (7). Data from all women in spontaneous labor at term in their first pregnancies and in which the babies were in a cephalic presentation were included. The outcome was emergency cesarean delivery. The registry contained a total of 32,868 eligible records, 3,817 (19.9%) of which had documented emergency cesarean deliveries. The 5 predictors used were maternal age, height, week of gestational age, infant sex, and birth weight percentile (corrected for sex and week of gestational age). We randomly created 150 small data sets, each containing 25 or 26 cases and 102 or 103 controls. Approval for the analyses was provided by the privacy advisory committee of the Information Services Division of National Health Service Scotland and by the Cambridge Local Research Ethics Committee 2.

### Assessment of optimism

All analyses were performed using Stata, version 12.1, software (StataCorp LP, College Station, Texas). The prediction models used for both clinical examples were logistic regression models, which included linear terms for continuous covariates and excluded interaction terms. We used the *C* statistic (the proportion of all possible pairwise combinations of cases and controls in which the case has a higher predicted probability of failure than the control) to assess predictive ability. The optimism of a model derived from a given small data set was assessed as follows. First, the model was fitted to all observations in the given small data set, and the *C* statistic was calculated, which we called the naïve *C* statistic. The model was then applied to a data set formed by pooling all of the other small data sets, and the *C* statistic was calculated, which we called the “true” *C* statistic. The difference between the naïve and true *C* statistics was the “optimism” for that small data set.

### Existing methods of adjustment of clinical data for optimism

In sample splitting, the small data set was randomly split into 2 separate groups of two-thirds and one-third. The predictive model was fitted to the data from the larger group, and the optimism-corrected estimate of the *C* statistic was calculated by applying this fitted model to the smaller group. Cross-validation was performed by randomly splitting the small data set into *k* equally sized groups. We used *k* = 10. Data on 1 group were excluded, and the model was fitted to the data on the other *k* − 1 groups. The resulting model was then applied to the excluded group, and the *C* statistic was calculated. This process was repeated *k* times, excluding each of the groups in turn. The resulting *k C* statistics were averaged to produce a single, overall optimism-corrected estimate of the *C* statistic. We also carried out cross-validation with replication. Here the cross-validation was replicated *r* times, with a different random split into *k* groups each time. It has been suggested that analyses should include development and testing of at least 200 models to generate an average (8). Hence, we used 20 replications of 10-fold cross-validation. The optimism-corrected estimate of the *C* statistic was the mean of the 200 values.

In leave-1-out cross-validation, a single observation was omitted from the small data set, and a model was fitted to the remaining observations and used to predict the probability of the outcome in the omitted observation. This process was repeated, omitting a different observation until all of the observations in the data set had an estimated probability calculated from a model fitted to all of the others. The *C* statistic was then calculated from these probabilities.

Bootstrapping was performed as described by Harrell et al. (3). The small data set was repeatedly resampled to produce *b* replicated data sets, each the same size as the original. We used *b* = 200. The predictive model was fitted to each of the *b* replicated data sets in turn. Each fitted model was then applied both to the resampled data set from which it was generated and to the original data set; the *C* statistic was calculated for both, and the difference between these 2 statistics was calculated. The *b* differences were then averaged to give an estimate of the optimism. The optimism-corrected estimate of the *C* statistic was then calculated as the naïve *C* statistic minus the estimated optimism.

### Leave-pair-out cross-validation

We developed a modification of cross-validation in which 1 case and 1 control were omitted from the small data set, a model was fitted to the remaining observations, and the fitted model was then used to predict the probability of the outcome in each member of the omitted pair. This process was repeated for every possible pairwise combination of case and control in the small data set. The *C* statistic was then calculated as the proportion of all pairwise combinations in which the predicted probability was greater for the case than for the control. We subsequently found that the method had previously been described in the machine learning literature, where it was called “leave-pair-out cross-validation” (9).

### Evaluating methods for optimism adjustment

All methods were performed in all of the small data sets. We quantified the systematic error of each method by subtracting the true *C* statistic, described above, from the optimism-corrected *C* statistic, calculated by the given method. Systematic error was inferred when the median signed difference for all of the small data sets was significantly different from 0. We also assessed the absolute error (i.e., the unsigned difference between the *C* statistic calculated by the given method and the true *C* statistic). The absolute error was calculated for all of the small data sets (50 for Down syndrome and 150 for cesarean delivery) using all of the methods. Then, for each small data set and for each pair of methods, we calculated the difference between the absolute errors. A pair of methods is inferred to have different absolute errors when the median (of the small data sets) difference in absolute error was significantly different from 0 using the Wilcoxon signed-rank test. The variability associated with repeated application of the same method to the same small data set was assessed by the standard deviation and range of 50 repeated analyses of a single representative small data set for each outcome.

### Simulation studies

To simulate a single data set resembling the original Down syndrome data set, we generated age, log maternal serum α-fetoprotein, and log maternal serum human chorionic gonadotropin values and the binary outcome variable for each of a large population of individuals and then sampled cases and controls from this population. Fifteen cases and 60 controls were selected to yield an EPV of 5 and a ratio of 4 controls per case. For each individual in the population, age was generated from a triangular distribution with minimum 15, maximum 47, and mode 31, and log maternal serum α-fetoprotein and log maternal serum human chorionic gonadotropin were generated from a bivariate normal distribution with mean dependent on age. These distributions were chosen after an exploratory analysis of the original Down syndrome screening data set. To generate the binary outcome, the logistic regression model estimated from the original data set was used but with coefficients multiplied by the same constant to ensure that the *C* statistic of this model was 0.90.

Likewise, to simulate a single data set resembling the original cesarean delivery data set, we generated data for each of a large population of individuals and then sampled 25 cases and 100 controls. For each individual in the population, we independently generated gestational age from a triangular distribution with minimum 37, maximum 42, and mode 39.5; maternal age from a uniform distribution with minimum 16 and maximum 37; and weight (measured as a centile) as uniform on 0–100. Height was normally distributed with mean depending on age, gestational age, and weight. Sex was generated by simulating a binary variable whose probability depended on the other 4 variables. These distributions were chosen after an exploratory analysis of the original data set. To generate the binary outcome, we used the logistic regression model estimated from the original data set, but with coefficients multiplied by the same constant to ensure that the *C* statistic was 0.71.

To modify the EPV, we scaled the numbers of cases and controls (e.g., 60 cases and 240 controls for an EPV of 20 in the data sets based on the Down syndrome study). The true area under the receiver operating characteristic curve was changed by scaling the coefficients in the logistic regression model for the binary outcome. In all simulations, we calculated the difference between the optimism-adjusted and true *C* statistics for 1,000 data sets for each outcome. We evaluated methods using the mean signed difference, the mean absolute (unsigned) difference, and the mean squared error.

## RESULTS

### Analysis of Down syndrome prediction

**Figure 1.**
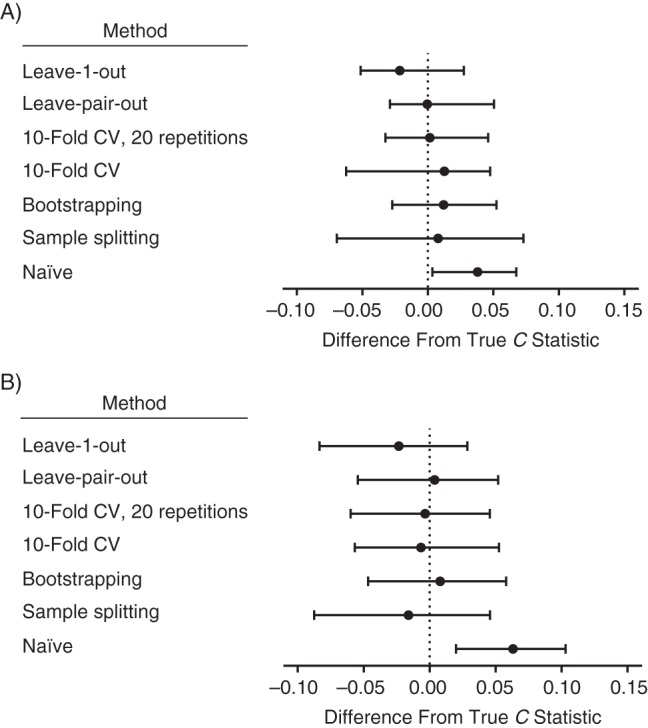
The medians and interquartile ranges of the difference between the *C* statistic estimated using different methods and the true *C* statistic for A) 50 small Down syndrome data sets and B) 150 small cesarean delivery data sets. The data are United Kingdom Down syndrome screening results from Glasgow (1991–2003), Edinburgh (1999–2003), and Cambridge (1990–2006), as well as Scottish national pregnancy discharge data (2004–2007)). Bars, 95% confidence intervals. CV, cross-validation.

**Figure 2.**
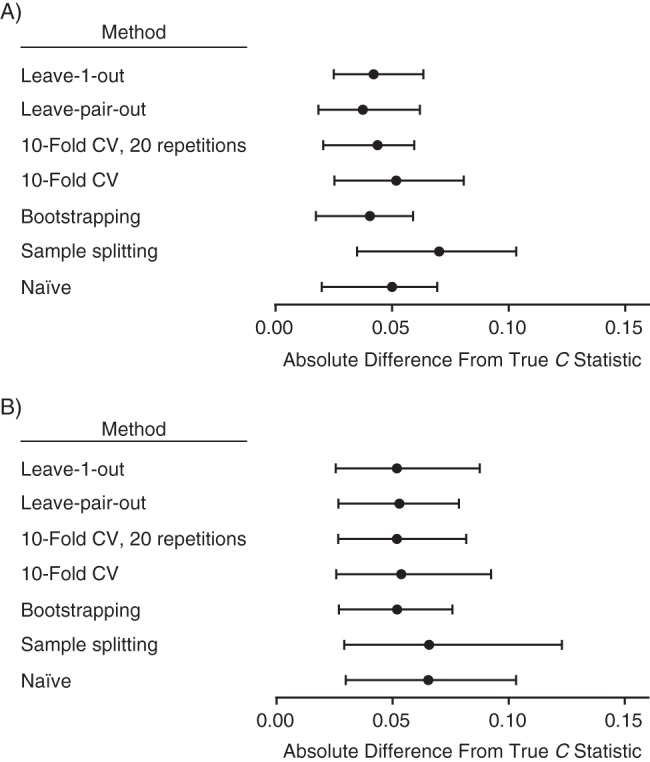
The medians and interquartile ranges of the absolute (unsigned) difference between the *C* statistic estimated using different methods of correcting for optimism and the true *C* statistic for A) 50 small Down syndrome data sets and B) 150 small cesarean delivery data sets. (See Table 1 for comparison of absolute errors comparing different methods.) The data are United Kingdom Down syndrome screening results from Glasgow (1991–2003), Edinburgh (1999–2003), and Cambridge (1990–2006), as well as Scottish national pregnancy discharge data (2004–2007)). Bars, 95% confidence intervals. CV, cross-validation.

The *C* statistic for the large data set (*n* = 4,111) was 0.901 (i.e., this is from a model fitted to the whole data set without correction for optimism). The mean of the naïve *C* statistics of the 50 small data sets was 0.915 (range, 0.761–0.993). The mean of the 50 true *C* statistics (defined above) was 0.886 (range, 0.817–0.903). The median and interquartile ranges of the difference between the true *C* statistic and the other methods for estimating the *C* statistic are plotted in Figure 1A. The naïve *C* statistic was overestimated; the *C* statistic corrected using leave-1-out cross-validation was systematically underestimated, but none of the other differences was significantly different from 0. The median and interquartile ranges of the absolute (unsigned) difference between the true *C* statistic and the methods for estimating the *C* statistic are plotted in Figure 2A. The pairwise differences in the *C* statistic comparing all the methods are tabulated in Table 1. Sample splitting, 10-fold cross-validation with no replications, and leave-1-out cross-validation all had greater absolute errors when compared with at least 1 of the other methods. There were no significant differences in the absolute errors of 10-fold cross-validation with 20 replications, bootstrapping, and leave-pair-out cross-validation.

**Table 1.** Pairwise Comparison of Absolute Errors Using Different Methods of Adjustment for Optimism Using Data on Down Syndrome and Cesarean Delivery, United Kingdom, 1990–2007

Method of Adjustment by Data Set	Median Difference in Absolute Error (IQR)^a^				
	Sample Splitting	Bootstrapping	10-Fold CV	10-Fold CV (20 Replications)	Leave-Pair-Out CV
Down syndrome data set					
Bootstrapping	0.022*** (–0.005–0.053)				
10-Fold CV	0.015 (–0.028–0.046)	–0.006** (–0.024–0.003)			
10-Fold CV (20 replications)	0.025*** (–0.008–0.0520)	0.002 (–0.006–0.007)	0.011*** (–0.003–0.025)		
Leave-pair-out CV	0.018*** (–0.005–0.052)	0.000 (–0.004–0.005)	0.007** (–0.004–0.024)	–0.002 (–0.005–0.003)	
Leave-1-out CV	0.014** (–0.006–0.053)	–0.003 (–0.031–0.016)	0.010 (–0.016–0.029)	–0.006 (–0.028–0.014)	–0.010* (–0.026–0.013)
Cesarean delivery data set					
Bootstrapping	0.012*** (–0.018–0.079)				
10-Fold CV	0.013** (–0.032–0.066)	–0.008** (–0.035–0.017)			
10-Fold CV (20 replications)	0.015*** (–0.023–0.069)	–0.001 (–0.014––0.008)	0.008 (–0.018–0.028)		
Leave-pair-out CV	0.015*** (–0.023–0.072)	0.000 (–0.009–0.006)	0.007* (–0.018–0.030)	–0.001 (–0.006–0.007)	
Leave-1-out CV	0.016** (–0.031–0.060)	–0.012** (–0.037–0.023)	0.000 (–0.026–0.029)	–0.009** (–0.029–0.016)	–0.019*** (–0.029–0.020)

Abbreviations: CV, cross-validation; IQR, interquartile range.

* *P* < 0.05, **P < 0.01, ***P < 0.001.

^a^ The absolute error associated with the method in the row is subtracted from the absolute error associated with the method in the column, and the medians and IQRs are presented for the 50 subgroups. Hence, positive values indicate greater absolute error using the method in the column, and negative values indicate lower absolute error. Statistical comparison is by the Wilcoxon signed-rank test versus the null hypothesis of no difference.

### Analysis of cesarean delivery prediction

**Figure 3.**
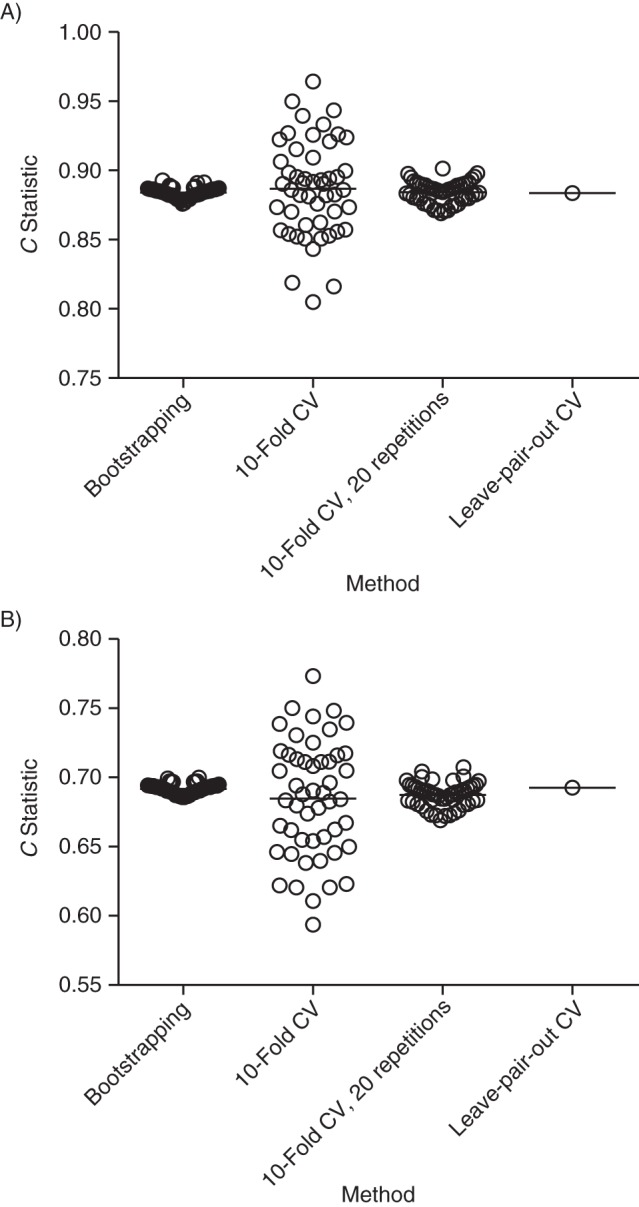
The *C* statistics for 50 consecutive analyses of a representative subsample illustrating the variability of repeated analysis using different methods. A) For the Down syndrome data, the standard deviations of the 50 repeated analyses were 0.004 (range, 0.876–0.893) for bootstrapping, 0.035 (range, 0.805–0.964) for 10-fold cross-validation (CV), and 0.008 (range, 0.869–0.901) for 10-fold cross-validation with 20 replications. B) For the cesarean delivery data, the standard deviations of the 50 repeated analyses were 0.004 (range, 0.686–0.700) for bootstrapping, 0.042 (range, 0.594–0.773) for 10-fold cross-validation, and 0.009 (range, 0.669–0.707) for 10-fold cross-validation with 20 replications. The data are United Kingdom Down syndrome screening results from Glasgow (1991–2003), Edinburgh (1999–2003), and Cambridge (1990–2006), as well as Scottish national pregnancy discharge data (2004–2007)).

The *C* statistic for the large data set (*n* = 19,215) was 0.711. The mean of the naïve *C* statistics from the 150 small data sets was 0.741 (range, 0.602–0.856). The mean of the 150 true *C* statistics was 0.681 (range, 0.581–0.709). The medians and interquartile ranges of the difference between the true *C* statistic and the methods for estimating the *C* statistic are plotted in Figure 1B. The naïve *C* statistic was overestimated, and the *C* statistics that were corrected using sample splitting and leave-1-out cross-validation were systematically underestimated, but none of the other medians was significantly different from 0. The median and interquartile ranges of the absolute (unsigned) difference between the true *C* statistic and the methods for estimating the *C* statistic are plotted in Figure 2B. The pairwise differences in the *C* statistic comparing all of the methods are tabulated in Table 1. Sample splitting, 10-fold cross-validation with no replications, and leave-1-out cross-validation all had greater absolute errors when compared with at least 1 of the other methods. There were no significant differences in the absolute errors of 10-fold cross-validation with 20 replications, bootstrapping, and leave-pair-out cross-validation. The variability of the estimates from repeated analyses of a representative small data set for each outcome is illustrated in Figure 3.

### Simulation studies

In both simulation studies, the signed difference was similar for bootstrapping and all forms of cross-validation in which the EPV was 10 or more, with the exception of the cesarean delivery simulation with a true *C* statistic of 0.61, for which bootstrapping was optimistic (Web Figure 1 available at http://aje.oxfordjournals.org/). When the EPV was 5 or less, bootstrapping tended to be optimistic, particularly when the *C* statistic was 0.71 or less. All forms of cross-validation tended to be pessimistic, although there was 1 simulation in which it was optimistic. The degree of pessimism associated with cross-validation was less than the degree of optimism with bootstrapping. Bootstrapping, 10-fold cross-validation with replications, and leave-pair-out cross-validation all had similar absolute errors when the EPV was 5 or more, or when the *C* statistic was 0.9 or greater (Web Figure 2). The absolute error was slightly lower with bootstrapping when the EPV was 2 and the *C* statistic was 0.61. The mean squared error was generally lower with bootstrapping when the EPV was 2, or when the EPV was 5 and the *C* statistic was less than 0.7 (Web Figure 3).

## DISCUSSION

When analyzing 2 clinical data sets, we found that 3 commonly used methods for correcting the *C* statistic for optimism performed poorly. Sample splitting generated results that were biased in the analysis of cesarean delivery prediction (Figure 1) and had greater absolute errors than any other method in both analyses (Figure 2). Ten-fold cross-validation produced unbiased estimates of the *C* statistic. However, in the cesarean delivery analysis, the absolute error in the *C* statistic was greater than with other methods. Leave-1-out cross-validation produced estimates that were both biased and had greater absolute errors than other methods in both analyses. We found that bootstrapping, 10-fold cross-validation with 20 replications, and leave-pair-out cross-validation all performed similarly in the clinical data sets, with unbiased estimates of the *C* statistic and comparable absolute errors.

In the simulation studies, we found that bootstrapping, 10-fold cross-validation with 20 replications, and leave-pair-out cross-validation all performed similarly when the EPV was 10 or greater or the *C* statistic was 0.9 or greater. When the EPV and *C* statistic were lower, the absolute and mean squared error tended to be lower with bootstrapping than with the different methods of cross-validation. However, when comparing the signed error, bootstrapping tended to be optimistic, and cross-validation (with replication and leave-pair-out) tended to be pessimistic, but the absolute magnitude of the bias tended to be lower for cross-validation than for bootstrapping. Hence, no single method clearly outperformed all of the other methods in terms of random and systematic errors.

When the different methods of cross-validation were compared, 10-fold cross-validation without replication was clearly inferior to the other methods, with much higher absolute and mean squared errors. Leave-pair-out cross-validation had signed and absolute errors that were similar to those of the other methods of cross-validation with replication. Our findings that it generated unbiased estimates of the *C* statistic with low absolute errors are similar to those of evaluations in the machine learning literature (9). Although the method has the advantage that it always generates the same value when applied repeatedly to the same data set, the far greater computational requirements are likely to limit its usefulness.

### The problem of optimistic prediction

The potential problem of optimistic prediction in multivariate models is well recognized (3). However, this issue is not dealt with in detail in some of the key literature. Many journals require that reports of new diagnostic tests conform to the Standards for the Reporting of Diagnostic Accuracy Studies guidelines (10). However, the guidelines do not require authors to address optimism. Moreover, methodological reviews about the development and validation of diagnostic tests did not address this issue in detail (11, 12). A systematic review of papers describing prognostic models for cancer using molecular markers found that correction for optimism was performed in only 3 of 129 articles (13). Moreover, when studies do correct for optimism, various methods are used. For example, predictive models for preeclampsia generated by recent (in the last 3 years) large-scale, multicenter, international, prospective cohort studies included correction using bootstrapping (14) and correction using 10-fold cross-validation with no replications (15).

### Why different methods perform differently

There are a number of issues that might explain differences between methods. A key aspect is uncertainty in the coefficients fitted to models and in the estimate of model performance. Both are present to a major degree in sample splitting, in which the model is more uncertain because of the exclusion of cases for validation, and the validation is more uncertain because of the exclusion of cases for generating the model. In cross-validation, the model is evaluated in all of the subjects. However, each model always includes a smaller sample size than the total, typically 90% (i.e., in the case of 10-fold cross-validation); hence, there remains more uncertainty in the coefficients than if the entire data set had been used. It follows, therefore, that cross-validation might be most effective when the number of subjects omitted when generating a given predictive model is lowest. However, leave-1-out cross-validation performed poorly. The issue here is that, although each omitted subject has an estimated probability derived from a model fitted to the whole of the rest of the data set, all of the models used to generate these probabilities were slightly different. Pooling probabilities from different models has previously been shown to result in biased (pessimistic) estimates of the *C* statistic (16), and we also found this in the current analysis. Leave-pair-out cross-validation has the advantage, therefore, that each model contains the largest possible number of subjects required to generate valid out-of-sample comparisons. However, there are still fewer subjects in any model than in bootstrapping, which uses resampling to increase the number of subjects to be the same as the complete data set.

A second issue that may explain different results is random sampling. Leave-pair-out cross-validation does not involve random sampling because it evaluates every possible pairwise combination of case and control for validation. In contrast, conventional cross-validation and bootstrapping both involve random sampling and, hence, no 2 analyses will yield identical results. It is recommended that analyses adjusting for optimism should include development and testing of at least 200 models (8), and this informed our choice of 20 replications of 10-fold cross-validation and 200 replications in bootstrapping. We found greater absolute errors when using cross-validation without replication than when using the other unbiased methods, and this likely reflects the greater random error, which is clearly illustrated in Figure 3. In contrast, cross-validation with 20 replications and bootstrapping with 200 samples showed much smaller degrees of variability between repeated analyses of the same small data set and, in both cases, the standard deviation of the *C* statistic was less than 0.01, which was small in relation to the absolute error (Figure 2). Hence, when we use these methods, most of the variability associated with estimating the *C* statistic reflects the inherent uncertainty of using a small data set rather than the variability associated with methods using random sampling. Nevertheless, the range of *C* statistics that was estimated across 50 analyses was approximately 0.02 for both 10-fold cross-validation with 20 replications and bootstrapping with 200 replications. Hence, studies using these methods should assess the variability of repeated analyses and should consider using greater numbers of replications.

Finally, we confirmed that the use of the naïve approach in small data sets resulted in biased (optimistic) estimation of the *C* statistic. This arises because the model is fitted to best describe the given data set. As studies become larger, this becomes less of an issue, because the potential for any single observation to have an important overall effect on any fitted model or on the evaluation of a fitted model diminishes with the size of the data set. In cross-validation, there is no potential for a single observation to have a major effect on either model development or validation, because there is no overlap between the subjects used for these processes in generating or validating a given model. In contrast, with bootstrapping, there is overlap between the 2 through the process of resampling. It is possible that this might explain the biased (optimistic) estimates of the *C* statistic in both simulation studies.

### Limitations of the present study

The number of models fitted for the leave-pair-out method increases with the size of the data set. It may therefore be computationally impractical in large data sets. We were able to perform 1,000 analyses of 60 cases and 240 controls (Down syndrome simulation with EPV = 20). Hence, it is practical in small data sets and, with further improvements in computational power, it is likely to become practical in large data sets in the future. In the present study, the number of cases per predictor was less than in other contexts (e.g., expression gene array). Further studies will be required to compare the performance of these methods in situations with larger numbers of predictors.

### Further work

There are a number of other aspects to building a predictive model when overfitting may be an issue, and further studies should compare the methods evaluated in the present analysis to address these issues. This could include the selection of variables, the inclusion of interaction terms, and the use of nonlinear transformations. It is possible that leave-pair-out cross-validation will perform less well in these roles, because all models share *n* − 2 observations, and the same variables (or transformations of variables, or interactions) will tend to be selected unless there is a single highly influential observation.

## Supplementary Material

Web Material
