# Supplementary material for: Correcting for Optimistic Prediction in Small Data Sets
Source: Am J Epidemiol. 2014 Jun 24;180(3):318–24. doi: 10.1093/aje/kwu140 (PMC4108045; doi:10.1093/aje/kwu140)
Supplement: Web Material [file supp_180_3_318__index.html]

Correcting for Optimistic Prediction in Small Data Sets — Web Material 

# Correcting for Optimistic Prediction in Small Data Sets

## Web Material

Web Material

**Files in this Data Supplement:**

- Web Material - Doc file
