# Supplementary material for: Correcting for Optimistic Prediction in Small Data Sets
Source: Am J Epidemiol. 2014 Jun 24;180(3):318–24. doi: 10.1093/aje/kwu140 (PMC4108045; doi:10.1093/aje/kwu140)
Supplement: Web Material [file supp_kwu140_kwu140supp.doc]

Web Material for

Smith GCS, Seaman SR, Wood AM, Royston P, White IR.

Correcting for optimistic prediction in small datasets.

Contents

Web Figure 1.

Web Figure 2.

Web Figure 3.

**Web Figure 1**

**

**

Mean (signed) error between optimism adjusted C statistic and true C statistic in 1000 datasets from simulation studies. Reps denotes replications, CV denotes cross validation, EPV denotes events per predictor. There are no values for leave pair out cross validation with an EPV of 20 due to a very prolonged analysis time.

**Web Figure 2**

**

**

Mean absolute (unsigned) error between optimism adjusted C statistic and true C statistic in 1000 datasets from simulation studies. Reps denotes replications, CV denotes cross validation, EPV denotes events per predictor. There are no values for leave pair out cross validation with an EPV of 20 due to a very prolonged analysis time.

**Web Figure 3**

**

**

Mean squared error between optimism adjusted C statistic and true C statistic in 1000 datasets from simulation studies. Reps denotes replications, CV denotes cross validation, EPV denotes events per predictor. There are no values for leave pair out cross validation with an EPV of 20 due to a very prolonged analysis time.
